# Host status of melon, carrot, and *Meloidogyne incognita*-susceptible and -resistant cotton, cowpea, pepper, and tomato for *M. floridensis* from California

**DOI:** 10.2478/jofnem-2024-0004

**Published:** 2024-03-14

**Authors:** Antoon T. Ploeg, Scott Edwards

**Affiliations:** Department of Nematology, University of California Riverside, 3401 Watkins Drive, Riverside, CA 92521, U.S.A

**Keywords:** California, *Meloidogyne floridensis*, *Meloidogyne incognita*, resistance, root-knot nematode, vegetables

## Abstract

The host status of carrot, melon, and susceptible and resistant cultivars of tomato, cotton, cowpea, and pepper for a California isolate of the peach root-knot nematode *Meloidogyne floridensis* was determined in greenhouse pot experiments. It was compared to a race 3 isolate of *M. incognita*. Melon was an excellent host for both isolates and roots were heavily galled after the 8-week trial. Carrot was a host for *M. incognita*, but a poor host for *M. floridensis*, although both isolates caused similar levels of galling. Susceptible cotton was a good host for *M. incognita* race 3, but a poor host for *M. floridensis*. Susceptible tomato, cowpea, and pepper were good hosts for both isolates. The *M. incognita* resistance in tomato and pepper was broken by *M. floridensis*. Resistant cowpea was a maintenance host as population levels of *M. floridensis* remained virtually unchanged over the trial period. We conclude that *M. floridensis* poses a risk to some important vegetable crops in California, as it reproduces on most vegetable crops, including some cultivars that are resistant to *M. incognita*. On susceptible crops, the reproduction of *M. floridensis* was always significantly less than that of *M. incognita*, and we hypothesize that in mixed species field populations, *M. incognita* will outcompete *M. floridensis*. This study demonstrates that efforts to limit the spread and prevent further introductions of *M. floridensis* in California are important to maintain the effectiveness of plant resistance as a nematode management strategy in vegetable crops.

Root-knot nematodes (RKN, *Meloidogyne* spp.) are economically important plant parasites with a broad host range (Luc et al., 1990; Castagnone-Sereno et al., 2013). The Southern root-knot nematode (*M. incognita*) is a common RKN in California, particularly in loamy and sandy soils. Host crops for this nematode include bell pepper (*Capsicum annuum*), carrot (*Daucus carota*), cantaloupe/honeydew melon (*Cucumis melo*), and tomato (*Solanum lycopersicum*) (Luc et al., 1990). All these are economically important crops in California. The state is the leading producer of each of these crops in the U.S.A., representing a combined acreage of 352,000 acres and a combined value of $2,232 million (CDFA, 2022). Cotton (*Gossypium barbadense/Gossypium hirsutum*) is grown on 113,00 acres in California with a value of $400 million (CDFA, 2022) and is a host to *M. incognita* race 3 and 4 populations (Taylor and Sasser, 1978). To limit crop damage and prevent RKN population build-up, host plant resistance can be an excellent management strategy. It reduces or eliminates the need for nematicides and does not require major changes in farming practices or purchasing special equipment. Host-plant resistance has been adopted on a large scale in California processing tomato production, and the vast majority of current cultivars are resistant to *M. incognita*, *M. javanica,* and *M. arenaria* (Roberts, 1992; Williamson, 1998). Although the resistance in all tomato cultivars is based on the presence of the single dominant *Mi*-1 gene (Kaloshian et al., 1996; Williamson, 1998) that has been employed for over 70 years, and resistance-breaking RKN populations occur in California and elsewhere (Williamson, 1998; Ploeg et al., 2023), it remains an effective management strategy (Castagnone-Sereno et al., 2007). Resistant bell pepper cultivars containing the *N*-gene conferring resistance to *M. incognita* (Thies et al., 2003) are available but have yet to be evaluated under commercial field conditions in California. *Meloidogyne incognita*-resistant cotton cultivars are available and are commercially grown (Ogallo et al., 1997; Ojeda-Rivera et al., 2022). A breeding program to develop *M. incognita*-resistant carrot cultivars is ongoing (Seo et al., 2014; Parsons et al., 2015; Roberts et al., 2016), but resistant carrot cultivars are not (yet) available. Similarly, all melon cultivars are susceptible to *M. incognita* (Nugent and Dukes, 1997). Although RKN resistant *Cucumis* species, particularly *C. metuliferus*, can be used as a rootstock for melon, they are not commercially available, which may negatively affect fruit quality (Siguenza et al., 2005; Exposito et al., 2020), and this strategy is not used in California melon production. Cowpea (*Vigna unguiculata*) is also highly susceptible to *M. incognita* (Roberts et al., 2005), and grain-type cultivars are grown as dry blackeye beans or peas in California on 3,500 acres (CDFA, 2022). However, different, vigorous growing, late blooming vining-type cultivars of cowpea are also of interest as a cover crop that can shade out weeds, fix nitrogen, and add a substantial amount of biomass to the soil (Aguiar et al., 2001; Roberts et al., 2005). A third type of cowpea (*Vigna unguiculata* subsp. *sesquipedalis*), known as long bean or asparagus bean, is grown in central California, mostly on small-scale diversified farms by Hmong growers for fresh consumption of the beans (Huynh et al., 2023). Cultivars of all three types of cowpea with *Rk*-gene resistance against *M. incognita* have been developed (Roberts et al., 2005; Huynh et al., 2023). They can be used to minimize nematode-associated yield loss (blackeye bean, long bean) and, when used as a cover crop, can also reduce soil nematode levels for a subsequent crop (Roberts et al., 2005; Huynh et al., 2023).

The peach root-knot nematode (*M. floridensis*) was found for the first time in California in 2018 from soil and root samples collected from stunted almond trees grafted onto a rootstock with resistance to the Southern root-knot nematode *M. incognita* in Merced County CA, USA (Westphal et al., 2019). Root samples collected from these resistant rootstocks exhibited galling symptoms typical for RKN infestation, and morphological and molecular analysis of RKN extracted from root and soil samples confirmed the presence of the peach root-knot nematode *M. floridensis* (Westphal et al., 2019). As a consequence of this first detection of *M. floridensis* from California, the nematode is now considered an A-rated pest, which means that any additional finds would also be subject to treatment and eradication efforts. Cultures of this nematode can only be kept in a USDA-certified quarantine facility (Chitambar, 2018). This nematode appears to have a very limited distribution in California, as subsequent preliminary sampling efforts have not resulted in positive findings. In other US states, the nematode has been reported from Florida (Brito et al., 2015), South Carolina (Reighard et al., 2019), and Georgia (Marquez and Hajihassani, 2023). The host range and morphology of *M. floridensis* appear similar to *M. incognita*. In the differential host test (Taylor and Sasser, 1978), the plants may respond similarly to *M. incognita* race-3 and *M. floridensis* (Marquez and Hajihassani, 2023). Consequently, *M. floridensis* was initially considered a race-3 isolate of *M. incognita* (Sherman and Lyrene, 1983; Handoo et al., 2004). However, a species-specific primer set (C2F3/M-Flor-R2) developed to amplify a short mitochondrial DNA fragment of *M. floridensis* has proven very useful in distinguishing this species from other common RKN species, i.e., *M. incognita, M. javanica*, and *M. arenaria* (Marquez and Hajihassani, 2023). Isolates of *M. floridensis* from Florida, South Carolina, California, and Georgia were genetically very similar (Marquez and Hajihassani, 2023). However, this was not reflected in the reproduction of different isolates on host plants. Limited data are available on the host status of vegetable crops for *M. floridensis*, and results differed among various studies or *M. floridensis* isolates. In multiple studies, tomato, eggplant, and watermelon were reported as hosts, and collards and peanuts as poor or non-hosts (Table 1). Cucumber and pepper were reported as a host or as poor/non-hosts depending on the study (Table 1). The reproduction factor RF (Pf/Pi = final population density/initial population density) was greater than 1 for *M. floridensis* on tomato cultivars containing the *Mi*-1 resistance gene in two of three studies (Table 1). The *N*-gene *M. incognita*-resistant pepper cultivar Charleston Belle was a poor host or a host for *M. floridensis* in the same study, depending on the *M. floridensis* isolate (Table 1).

**Table 1:** Reported host status and reproduction factor (RF) of vegetable crop cultivars for *Meloidogyne floridensis*.

**Crop**	**Cultivar^a^**	**Host status^b^**	**RF**
collards^c^	NA	−	<1
collards^d^	Flash	−	0.6
cowpea^d^	Iron Clay	+	7.1
cucumber^d^	Mongoose	+	18.3
cucumber^c^	NA	−	<1
cucumber^e^	NA	+	NA
eggplant^c^	NA	+	8.1
eggplant^e^	NA	+	NA
peanut^f^	Florunner	−	NA
peanut^g^	Florunner	−	NA
pepper^f^	Keystone resistant giant	+	0.5–1.5
pepper^g^	California Wonder	−	NA
pepper^f^	California Wonder	+	1.0–5.2
pepper^c^	NA	−	<1
pepper^f^	Charleston Belle (*N*-gene)	+	0.3–1.2
snap bean^e^	NA	+	NA
squash^c^	NA	+	7.6
sweet potato^h^	Beauregard	+	31.1
sweet potato^h^	Bellevue	−	0.1
sweet potato^h^	Bonita	+	2.9
sweet potato	Burgundy	−	0.0
sweet potato^h^	Covington	−	0.0
sweet potato^h^	Diane	+	3.7
sweet potato^h^	Murasaki-29	+	4.8
tomato^d^	Rutgers	+	18.7–73.2
tomato^f^	Rutgers	+	NA
tomato^g^	Rutgers	+	NA
tomato^f^	Talladega	+	2.9–5.5
tomato^h^	Daniela	+	72.0
tomato^c^	NA	+	1.9
tomato^e^	NA	+	NA
tomato^h^	Celebrity (*Mi*-1 gene)	+	2.7
tomato^d^	Skyway (*Mi*-1 gene)	+	10.9–49.4
tomato^f^	Crista (*Mi*-1 gene)	−	0.2–0.6
watermelon^d^	Hybrid Crimson	+	4.0
watermelon^f^	Charleston Grey	+	NA
watermelon^g^	Charleston Grey	+	NA

a NA: not available

b −: reproduction factor < 1 or reported as resistant or non-host. +: reproduction factor ≥ 1 or reported as a host plant

c Kokalis-Burelle and Nyczepir, 2004

d Marquez and Hajihassani, 2023

e Brito et al., 2008

f Stanley et al., 2009

g Handoo et al., 2004

h Ploeg and Stoddard, 2023

The California isolate of *M. floridensis* was reported to break resistance in *Mi*-1 gene carrying tomato and in some sweet potato cultivars resistant to *M. incognita* (Ploeg and Stoddard, 2023). Still, it is unknown if this isolate can also break resistance in other vegetable crops. In this study, we compared the reproduction and root-galling of the California isolate of *M. floridensis* to an *M. incognita* race-3 isolate on *M. incognita*-susceptible and -resistant tomato, pepper, and cowpea. Although no resistant cultivars are available, melon and carrot were included because their host status for *M. floridensis* is unknown, and they are important RKN-susceptible vegetable crops in California. Cotton was included as an important non-vegetable crop with RKN-resistant cultivars available.

## Materials and methods

*Meloidogyne isolates*: The isolation, culturing, and identification of the California *Meloidogyne floridensis* isolate (*Mflor*) and the California *M. incognita* race-3 isolate (*Minc-3*) used in this study was as described previously (Ploeg et al., 2023; Ploeg and Stoddard, 2023). In short, *M. floridensis* was isolated from infected galled almond rootstock, and a single-egg-mass isolate was multiplied and cultured on tomato Daniela plants in a greenhouse inside the Nematode Quarantine Facility at the University of California Riverside under CDFA permit 398. The greenhouse temperature ranged between 21–26 °C, and plants were grown under natural light and fertilized with 5 g of Osmocote 17–6–10 controlled release fertilizer once every 8 weeks (Scotts-Sierra Horticultural Products Co, Marysville, OH). Species identification was done by PCR (Westphal et al., 2019) using second-stage juveniles that were hatched from the egg suspension used as inoculum. The *M. incognita* race-3 isolate (*Minc-3*) was originally isolated from cotton in the San Joaquin Valley, California, and has been maintained on susceptible tomatoes in our greenhouse for several years. Species identification was confirmed through PCR as described by Dong et al. (2001) using primers FMi 5′-CTC TGC CCA ATG AGC TGT CC-3′ and RMi 5′-CTCTGCCCTCACATTAAG-3′. Eggs of *Mflor* and *Minc-3* used as inoculum in the experiments were extracted from roots of tomato Daniela by shaking for 3 min in a 0.5% NaOCl solution (Hussey and Barker, 1973) and collected by washing over two stacked 25 μm pore-size sieves.

*RKN reproduction on selected crop cultivars:* Root-galling and multiplication of *Mflor* and *Minc-3* on *M. incognita*-susceptible tomato cultivar Daniela (Osborne Quality Seeds, Mt. Vernon, WA), pepper cultivar Baron (Seminis, Oxnard, CA), cotton cultivar SJ2 (originally obtained from California Planting Cotton Seed Distributors and propagated in our greenhouse), cowpea cultivar CB46 Null-1 (seed kindly provided by Dr. Huynh, Dept. Nematology, UC Riverside), carrot cultivar Imperator 58 (Mountain Valley Seed, Salt Lake City, UT), and melon cultivar Durango (Seminis, Oxnard, CA) and on *M. incognita*-resistant tomato cultivar Celebrity (Bayer/Seminis, St. Louis, MO), pepper cultivar Carolina Wonder (Reimer Seeds, Saint Leonard, MD), cotton cultivar DP 1747NR B2XF (seed kindly provided by Dr. Wheeler, Texas A&M), and cowpea cultivar CB46 (seed kindly supplied by Dr. Huynh, Dept. Nematology, UC Riverside) was determined in a replicated quarantine-greenhouse pot study. Tomato and pepper were seeded in a multicell seedling tray with potting mix (Sunshine Mix 5, Sungro, Vancouver, BC, Canada) and placed in a greenhouse. Three (tomato) or four (pepper) weeks after emergence, plants were carefully removed from the seedling trays, and roots gently washed free of potting mix and transplanted into 1-liter white plastic cups filled with steam-sterilized sand (93% sand, 4% silt, 3% clay, pH 7.1). Cotton, carrot, cowpea, and melon were seeded directly in 1-liter white plastic cups filled with steam-sterilized sand (three seeds per cup) and thinned to leave one plant per cup one week after emergence. Ten pots were set up for each crop cultivar, and five pots were each inoculated with 10 ml of a suspension containing 1,000 eggs/ml of *Mflor* and five pots with *Minc-3* by adding 2.5 ml to each of four holes around the roots of each plant (10,000 eggs per plant). All pots were inoculated at the same time (tomato and pepper 1 week after transplanting, carrot 4 weeks after emergence, cotton 3 weeks after emergence, cowpea and melon 2 weeks after emergence). The pots were randomized over a metal galvanized greenhouse bench, allowing free water draining. They were watered daily with a slow drip to avoid water/soil splashing contamination among pots through an automated drip-irrigation system and fertilized one week after inoculation with 5 g of Osmocote 17–6–10 controlled release fertilizer (Scotts-Sierra Horticultural Products Co, Marysville, OH). The greenhouse temperature ranged between 21–26 °C under natural light. Plants were removed after 8 weeks, and the roots were washed free of soil. They were rated for the presence of root galling (0–10 scale: 0=no galling, 10=100% of root system galled. Bridge and Page, 1980). Eggs were extracted from the roots by shaking for 3 min in a 0.5% NaOCl solution (Hussey and Barker, 1973), collected by washing over two stacked 25 μm pore-size sieves, and counted under a dissecting microscope at 40x magnification. The complete experiment was repeated once.

The effect of the two RKN isolates on root-galling and egg production was analyzed separately for each plant cultivar. Prior to analysis, egg counts were transformed by x^1^ = log_10_(x+1) to standardize the variance. The significance of the RKN isolate, the repeated experiment (1 or 2), and the interactive effect on egg production was determined using the ANOVA procedure in SAS statistical software. Where treatment effects were significant at the 95% confidence level, treatment means were compared using Fisher's LSD procedure also at the 95% confidence level. Effects on the average galling index were analyzed with the non-parametric Kruskall-Wallis test (SAS Institute, Cary, N.C.).

## Results

The interactive effect of “experiment × RKN isolate” on the degree of root galling was significant (P < 0.05) for melon, the susceptible tomato, the resistant pepper, and the susceptible and resistant cowpea. Therefore, the results are shown separately for the two experiments (Table 2).

**Table 2:** Average (n=5 for each crop cultivar - *Meloidogyne* isolate combination) root-galling index (0–10 = no galling – 100% of root system with galls; ±standard error) on roots of different plant cultivars 8 weeks after inoculation with 10,000 eggs/plant of a California isolate of *M. incognita* race 3 (*Minc-3*) or *M. floridensis* (*Mflor*) in two repeated greenhouse pot experiments.

**Crop**	**Cultivar**	**Experiment**	***Meloidogyne isolate***			
	
			***Minc-3***		***Mflor***	
Tomato	Daniella (S)^a^	1	7.6±0.2	a^b^	7.4±0.2	a
		2	8.0±0.3	a	6.2±0.5	b
	Celebrity (R)	1	0.4±0.4	b	2.2±0.2	a
		2	0.8±0.2	b	1.8±0.4	a
Cotton	SJ2 (S)	1	2.4±0.2	a	2.6±0.2	a
		2	3.6±0.2	a	2.2±0.4	b
	DP 1747NR B2XF (R)	1	2.0±0.3	a	1.0±0.4	a
		2	1.2±0.2	a	1.2±0.2	a
Cowpea	CB46Null (S)	1	5.4±0.2	b	6.8±0.4	a
		2	6.0±0.3	a	4.8±0.7	a
	CB46RR (R)	1	0.0±0.0	a	0.0±0.0	a
		2	0.0±0.0	b	1.0±0.3	a
Pepper	Baron (S)	1	4.2±0.6	a	2.6±0.2	b
		2	4.0±0.5	a	1.0±0.3	b
	Carolina Wonder (R)	1	0.0±0.0	b	2.0±0.0	a
		2	0.0±0.0	b	1.0±0.1	a
Carrot	Imperator58 (S)	1	5.8±0.9	a	4.4±0.5	a
		2	5.8±0.9	a	4.8±0.4	a
Melon	Durango (S)	1	9.0±0.1	a	8.0±0.1	b
		2	8.5±0.2	a	8.4±0.2	a

a (S) or (R) indicates a *Meloidogyne incognita* susceptible or resistant cultivar, respectively.

b Different letters within rows indicate significant differences at the 95% confidence level, Kruskal-Wallis test.

Roots of melon Durango and the susceptible tomato Daniela were heavily galled, and roots of the resistant cowpea CB46RR remained virtually free of galling after inoculation with either *Meloidogyne* isolate. *Mflor* resulted in consistently higher root galling than *Minc-3* on the resistant tomato Celebrity and the resistant pepper Carolina Wonder. However, the reverse was true with *Minc*-3 on the susceptible pepper Baron, resulting in more severe galling than *Mflor*.

Statistical analysis of the nematode reproduction x^1^ = [log_10_(eggs per root system + 1)] showed that the “experiment *x* RKN isolate” interactive effect was only significant on the susceptible cowpea CB46Null (results not shown). However, as the separation of the means was the same between the two experiments, the results are presented as the average of 10 replicates.

The host status of a crop cultivar for RKN can be categorized according to the RF of the nematode on that plant as a good host (RF ≥ 1), a poor host (0.1 < RF <1), or a non-host (RF ≤ 0.1) (Oostenbrink, 1966; Sasser et al., 1984). The reproduction factor RF was significantly different between *Mflor* and *Minc-3* on each of the tested crop cultivars (Table 3). On the susceptible crop cultivars tomato Daniela, cowpea CB46Null, pepper Baron, and melon Durango, RF values for *Minc-3* were significantly higher than for *Mflor*, but as RF values on all these crop cultivars were much greater than 1 (RF values: 6.3 – 57.2), they can be considered as good hosts for both RKN isolates (Table 3). The resistance of cowpea CB46RR, pepper Carolina Wonder, and tomato Celebrity to *Minc-3* was very strong, with RF values of 0.1, 0.1, and 0.15, respectively. However, *Mflor* was able to overcome the resistance in tomato and pepper, instead increasing 1.8-fold and 8.2-fold, respectively, on these resistant crop cultivars (Table 3). The resistance in cowpea to *Mflor* was slightly compromised, and population levels on this crop stayed approximately even over the duration of the trial (RF = 0.9). Both cotton SJ2 and carrot Imperator 58 were good hosts for *Minc-3* but poor hosts for *Mflor* (Table 3). Surprisingly, this difference was not reflected in root-galling (Table 2).

**Table 3:** Average (n=10 for each crop cultivar - *Meloidogyne* isolate combination) root-knot nematode reproduction factor RF (number of eggs per root system at harvest/number of eggs inoculated; ±standard error) on different plant cultivars 8 weeks after inoculation with 10,000 eggs/plant of a California isolate of *M. incognita* race 3 (*Minc-3*) or *M. floridensis* (*Mflor*) in greenhouse pot experiments.

**Crop**	**Cultivar**	***Meloidogyne isolate***			

		***Minc-3***		***Mflor***	
Tomato	Daniella (S)^a^	50.6±5.5	a^b^	18.9±2.4	b
	Celebrity (R)	0.15±0.1	b	1.8±0.4	a
Cotton	SJ2 (S)	5.8±0.6	a	0.7±0.1	b
	DP 1747NR B2XF (R)	0.8±0.2	a	0.1±0.0	b
Cowpea	CB46Null (S)	57.2±5.3	a	37.0±6.0	b
	CB46RR (R)	0.1±0.0	b	0.9±0.2	a
Pepper	Baron (S)	55.4±9.7	a	6.3±1.5	b
	Carolina Wonder (R)	0.1±0.0	b	8.2±0.7	a
Carrot	Imperator58 (S)	3.6±0.5	a	0.7±0.1	b
Melon	Durango (S)	18.0±1.9	a	9.5±1.5	b

a (S) or (R) indicates a *Meloidogyne incognita* susceptible or resistant cultivar, respectively.

b Statistical analysis was done on log(x+1)-transformed data. Non-transformed data shown. Different letters within rows indicate significant differences at the 95% confidence level (Fisher's protected LSD test).

## Discussion

This is the first report on the host status of carrot and melon for *M. floridensis*. Carrot has been reported in many studies as a good host for four common RKN species: *M. incognita*, *M. javanica*, *M. arenaria*, and *M. hapla* (Ferris, 2023). Race 1 isolates of the economically important Columbia RKN *M. chitwoodi* reproduce on carrots and can be distinguished from race 2 isolates, which do not reproduce on this crop (Mojtahedi et al., 1988; Santo et al., 1988). In this study, carrot was a poorer host for *M. floridensis* (RF = 0.7) than for *M. incognita* (RF = 3.6). According to these RF values, carrot is designated as a poor host for *M. floridensis* and as a good host for *M. incognita*. Whether large differences exist in the host status of carrots for different isolates of *M. floridensis* remains to be studied. Interestingly, the ability to induce galling on carrots was not different between *M. floridensis* and *M. incognita*. Therefore, it is likely that growing carrots on fields infested with *M. floridensis* will impact carrot quality similarly to *M. incognita*. Like carrot, melon is also a good host for the most common RKN species (Ferris, 2023). In California, *M. incognita* is the most important RKN species on melon, resulting in stunting, yield loss, and severe root galling (Radewald et al., 1975). In this study, melon was an excellent host for both *M. incognita* and *M. floridensis*, although reproduction of the former was higher (RF 18.0 vs. RF = 9.5). Melon roots were severely galled after inoculation with either RKN isolate. Results on tomato corresponded with our previous results (Ploeg and Stoddard, 2023). The susceptible Daniela is an excellent host for both RKN isolates and the *Mi*-gene resistant Celebrity is resistant to *M. incognita* but susceptible to *M. floridensis*. The ability of *M. floridensis* to break *Mi*-gene resistance in tomato also agrees with previous results (Stanley et al., 2009; Marquez and Hajihassani, 2023). Tomato root-galling caused by *M. floridensis* was light but still significantly higher than after *M. incognita* inoculation. It can, therefore, be concluded that *Mi*-gene-carrying tomato cultivars allow the reproduction of *M. floridensis* and that attempts to use host plant resistance as a management strategy to lower *M. floridensis* population levels will probably prove ineffective in tomatoes. Cotton SJ2 was a good host for the *M. incognita* isolate used in this study. This was expected as the isolate was a race 3, able to reproduce on cotton (Taylor and Sasser, 1978). Handoo et al. (2004) reported that cotton was slightly infected and rated it a poor host for *M. floridensis*. This agrees with our results, as cotton was infested, and the roots developed slight galling, but the population declined (RF = 0.7). The resistant cotton DP 1747NR B2XF was resistant to *M. incognita* and highly resistant to *M. floridensis*, and using RKN-resistant cotton promises to be an effective method for lowering both *M. incognita* and *M. floridensis* populations. The susceptible cowpea CB46Null was reported as a good host for *M. incognita* and *M. javanica* (Ndeve et al., 2018). It was an excellent host for both RKN isolates in our study, resulting in a 57-fold and 37-fold increase in nematode levels for *M. incognita* and *M. floridensis*, respectively. The designation of cowpea as a good host for *M. floridensis* by Marquez and Hajihassani (2023) is confirmed by our results. The previously established strong resistance of the *Rk*-gene-carrying cowpea CB46 to *M. incognita* (Ndeve et al., 2018; Ploeg et al., 2023) agrees with our results. However, the resistance of this cowpea cultivar was partially overcome by *M. floridensis*, which infected but did not increase (RF = 0.9). It caused very minor root-galling on this cultivar, which should be considered a poor host for *M. floridensis*. Examples of very minimal root-galling coinciding with RKN reproduction on cowpea and Lima beans were observed previously by Ndeve et al. (2018) and Roberts et al. (2008). They determined that in these crops, different genetic factors were involved in resistance to galling and nematode reproduction. It remains to be determined if a similar mechanism can explain the response of cowpea CB46 to *M. floridensis*. Pepper Baron was a good host for both isolates, although the reproduction of *M. incognita* was much higher than that of *M. floridensis*. Galling of the pepper roots was not very strong, even after inoculation with *M. incognita,* which reached very high egg densities. Previous reports on the host status of pepper for *M. floridensis* are contradictory, with some studies concluding that pepper was a non-host (Handoo et al., 2004; Kokalis-Burelle and Nyczepir, 2004), while others reported pepper as a host (Stanley et al., 2009). Variability between isolates of *M. floridensis* or differences in experimental conditions or between pepper cultivars used may be the reason for the reported differences in the host status of pepper for *M. floridensis*. The *N*-gene resistance in pepper Carolina Wonder was very effective against *M. incognita* but ineffective against *M. floridensis*. Stanley et al. (2009) reported RF values ranging between 0.3 and 1.2 of four *M. floridensis* isolates from Florida on the *N*-gene resistant pepper Charleston Belle. They concluded that this pepper cultivar was susceptible to two and resistant to two isolates but that even the two poorly reproducing isolates were able to overcome the *N*-gene resistance. Compared to their results, the results from our experiments are much less ambiguous as the *N*-gene-carrying Carolina Wonder can be designated as a good host for *M. floridensis*.

Interestingly, the reproduction of *M. incognita* was significantly higher than that of *M. floridensis* on all the susceptible crop cultivars. This would imply that, under temperatures similar to those in our experiments, a field population consisting of a mixture of *M. incognita* and *M. floridensis* under a susceptible crop may, over time, shift to a larger proportion of the population belonging to *M. incognita*. In our study, pepper Carolina Wonder was a suitable host to distinguish *M. incognita* from *M. floridensis*. Other hosts that consistently separated *M. floridensis* from *M. incognita* include several *M. incognita*-resistant *Prunus* rootstocks such as NemaGuard, NemaRed, FlordaGuard, Guardian, Hansen 536, and Bright's Hybrid 5 (Handoo et al., 2004; Stanley et al., 2009; Westphal et al., 2019).

In conclusion, our results show that *M. floridensis* is able to overcome resistance in pepper, tomato, and, to a lesser extent, cowpea and that some important California vegetable crops are a host for this nematode (Table 4).

**Table 4:** Summary of results from this study on host status and reproduction factor (RF) of vegetable crop cultivars for *Meloidogyne floridensis*.

**Crop**	**Cultivar**	**Host status^a^**	**RF**
carrot	Imperator58	−	0.7
cotton	SJ2	−	0.7
cotton	DP 1747NR B2XF (*RK1* gene)	−	0.1
cowpea	CB46Null	+	37.0
cowpea	CB46RR (*Rk* gene)	−	0.9
melon	Durango	+	9.5
pepper	Baron	+	6.3
pepper	Carolina Wonder (*N* gene)	+	8.2
tomato	Daniella	+	18.9
tomato	Celebrity (*Mi*-1 gene)	+	1.8

a −: reproduction factor < 1. +: reproduction factor ≥ 1.

The nematode is relevant to California vegetable production, as it limits resistance-based management options, and consequently, continuing and future efforts to contain, eradicate, and limit the further distribution or new introductions of this nematode are important.
